# Annexin A1 N-Terminal Derived Peptide Ac2-26 Stimulates Fibroblast Migration in High Glucose Conditions

**DOI:** 10.1371/journal.pone.0045639

**Published:** 2012-09-21

**Authors:** Valentina Bizzarro, Bianca Fontanella, Anna Carratù, Raffaella Belvedere, Raffaele Marfella, Luca Parente, Antonello Petrella

**Affiliations:** 1 Department of Pharmaceutical and Biomedical Sciences, University of Salerno, Salerno, Italy; 2 Telethon Institute of Genetics and Medicine (TIGEM), Naples, Italy; 3 Department of Geriatrics and Metabolic Diseases, Second University of Naples, Naples, Italy; Tohoku University, Japan

## Abstract

Deficient wound healing in diabetic patients is very frequent, but the cellular and molecular causes are poorly defined. In this study, we have evaluated whether Annexin A1 derived peptide Ac2-26 stimulates fibroblast migration in high glucose conditions. Using normal human skin fibroblasts WS1 in low glucose (LG) or high glucose (HG) we observed the enrichment of Annexin A1 protein at cell movement structures like lamellipodial extrusions and interestingly, a significant decrease in levels of the protein in HG conditions. The analysis of the translocation of Annexin A1 to cell membrane showed lower levels of Annexin A1 in both membrane pool and supernatants of WS1 cells treated with HG. Wound-healing assays using cell line transfected with Annexin A1 siRNAs indicated a slowing down in migration speed of cells suggesting that Annexin A1 has a role in the migration of WS1 cells. In order to analyze the role of extracellular Annexin A1 in cell migration, we have performed wound-healing assays using Ac2-26 showing that peptide was able to increase fibroblast cell migration in HG conditions. Experiments on the mobilization of intracellular calcium and analysis of p-ERK expression confirmed the activity of the FPR1 following stimulation with the peptide Ac2-26. A wound-healing assay on WS1 cells in the presence of the FPR agonist fMLP, of the FPR antagonist CsH and in the presence of Ac2-26 indicated that Annexin A1 influences fibroblast cell migration under HG conditions acting through FPR receptors whose expression was slightly increased in HG. In conclusion, these data demonstrate that (i) Annexin A1 is involved in migration of WS1 cells, through interaction with FPRs; (ii) N- terminal peptide of Annexin A1 Ac2-26 is able to stimulate direct migration of WS1 cells in high glucose treatment possibly due to the increased receptor expression observed in hyperglycemia conditions.

## Introduction

Diabetes mellitus is a group of metabolic disorders that causes chronic hyperglycemia and is one of the most significant diseases in the developed world [1]. The inadequate treatment of hyperglycemia leads to severe complications in diabetic patients, including impaired wound healing, that cause long-term complications such as limb amputation [2].

Skin wound repair involves a series of coordinated processes that include cell proliferation and migration, collagen deposition and remodeling, wound contraction, and angiogenesis. Different cell types are involved in this process including fibroblasts/myofibroblasts, keratinocytes, and endothelial cells [3], [4]. Some studies have described alterations in cell migration associated with diabetic conditions. It has been shown [5] that fibroblasts from diabetic mice migrate 75% less than those from normoglycemic mice and display a defective response to hypoxia, a condition commonly present in chronic wounds. A similar inhibition was observed in keratinocytes cultured in a high glucose environment [6]. More recently it has been shown that hyperglycemia impairs cell migration through increased generation of ROS, which induces an abnormal activation of Rac1, confirming that high glucose plays a direct role on cell migration [7].

Annexin A1 (ANXA1, lipocortin-1) is the first characterized member of the annexin superfamily of proteins, so called since their main property is to bind (i.e., to annex) to cellular membranes in a Ca^2+^-dependent manner. ANXA1 has been involved in a broad range of molecular and cellular processes, including anti-inflammatory signaling, kinase activities in signal transduction, maintenance of cytoskeleton and extracellular matrix integrity, tissue growth, apoptosis and differentiation [8]. The ANXA1 receptors on leukocytes have been identified as members of the formyl peptide receptor (FPR) family [9].

FPR, the founding member of the family, is a G-protein-coupled chemoattractant receptor, which can sense gradients of bacterial peptides of the prototype formylmethionineleucinephenylalanine (fMLP) and thereby directs leukocytes towards sites of bacterial infection [10]. Following ligand binding, FPRs undergo a conformational change that enables them to interact with the G proteins, possibly of the Gi and Gq family. FPR ligation has also been shown to signal through the small G protein Cdc42 to activate Rac and ARP2/3-dependent pathways leading to actin nucleation [11]. It has been demonstrated that FPRs are expressed in normal human lung, skin fibroblasts and human fibrosarcoma cell line HT-1080 and that the stimulation with fMLP triggers dose-dependent migration of these cells. Furthermore, fMLP is able to induce signal transduction including intracellular calcium flux and a transient increase in F-actin [11].

ANXA1 has been shown to regulate leukocyte migratory events through interactions with nFPRs. Previously, it has been identified the expression of functional *N*-formyl peptide receptors in model SK-CO15 intestinal epithelial cells and observed a role for activation of these receptors in regulating cellular invasive behavior [12]. We have recently shown that endogenous ANXA1 positively modulates myoblast cell differentiation by promoting migration of satellite cells [13].

Extensive research has focused on general cell migration and it is evident that the central mechanism underlying this phenomenon is the dynamic reorganization of the actin cytoskeleton. It was shown that ANXA1 accumulates together with F-actin at level of cell movement structures like lamellipodia and phillopodia. The bond between ANXA1 and actin that is observed *in vitro* seems to be related to ANXA1 co-localization with F-actin at plasma membrane ruffle levels induced by EGF [14], [15]. It’s therefore likely that the binding of ANXA1 to FPRs could lead to protein accumulation at leading edge of the cells in a Ca^2+^-dependent manner amplifying the pathway downstream FPR activation and contributing to actin polymerization.

Impaired fibroblast migration may contribute to the poor wound healing observed in diabetic patients and may represent a target of therapeutic intervention. Therefore, in this study we addressed the role of ANXA1 on fibroblast cell migration in high glucose treatment.

We show in skin fibroblast cell line WS1 that ANXA1 is involved in the processes of cell migration through interaction with FPRs, both in normal and hyperglycemic conditions. More importantly, the N-terminal peptide of ANXA1, Ac2-26 was also able to stimulate direct migration of WS1 cells in high glucose treatment.

## Materials and Methods

### Cell Culture

WS1, normal human skin fibroblast cells (ATCC, Rockville, MD, USA) were cultured in Minimum Essential Medium Eagle (EMEM; Lonza) containing L-Glutamine 2 mM supplemented with antibiotics (10000 U/ml penicillin and 10 mg/ml streptomycin; Lonza), 10% of heat-inactivated fetal bovine serum (FBS; Lonza) and 1% of non essential amino acids (NEAA; Lonza). Cultures destined for immunohistochemistry were grown to dense confluency on glass coverslips. High glucose conditions were reached by adding to the culture medium 25 mM of D-Glucose (Sigma Aldrich) for a minimum of 3 days before any additional treatment. The appropriate concentrations of the peptide were chosen following a dose-effect curve: 100 nM, 1 uM, 10 uM and 30 uM with Ac2-26 inducing effects on WS1 cells starting from 1 uM to 10 uM.

### Western Blot Analysis

Details of the procedure for immunoblotting have been previously described [16]. After three washing in TBST, the blots were incubated overnight at 4°C with primary polyclonal antibody against ANXA1 (1∶10000; Invitrogen), with primary polyclonal antibody against p-ERK (1∶1000; Cell Signaling), with primary monoclonal antibody against α-tubulin (1∶2000; Sigma-Aldrich) and then at RT with an appropriate secondary rabbit or mouse antibody (1∶5000; Sigma-Aldrich). Immunoreactive protein bands were detected by chemioluminescence using enhanced chemioluminescence reagents (ECL) and exposed to Hyperfilm. The blots were scanned and analyzed (Gel-Doc 2000, BIO-RAD). All results are mean ± SEM of 3 or more experiments performed in triplicate. The optical density of the protein bands detected by Western blotting was normalized on tubulin levels.

Statistical comparison between groups were made using Bonferroni parametric test. Differences were considered significant if p<0.01.

### Confocal Microscopy

After the specific time of incubation, WS1 cells were fixed in p-formaldehyde (4% v/v in PBS) for 5 minutes. The cells were permeabilized in Triton X-100 (0.5% v/v in PBS) for 5 minutes, and then incubated in goat serum (20% v/v PBS) for 30 minutes, and with a rabbit anti-ANXA1 antibody in PBS (1∶100; Invitrogen) overnight at 4°C. After two washing steps with PBS, cells were incubated with AlexaFluor anti-rabbit (1∶1000; Molecular Probes) for 2 h, and FITC-conjugated anti-F-actin (Phalloidin-FITC, Sigma) antibody for 30 minutes. The coverslips were mounted in glycerol (40% v/v PBS). A Zeiss LSM 710 Laser Scanning Microscope (Carl Zeiss MicroImaging GmbH Jena Germany) was used for data acquisition. To detect nucleus and filaments, samples were excited with a 458 and 488 nm Ar laser respectively. A 555 nm He-Ne laser was used to detect emission signals from ANXA1 stain. Samples were vertically scanned from the bottom of the coverslip with a total depth of 5 µm and a 63X (1.40 NA) Plan-Apochromat oil-immersion objective. A total of 10 z-line scans with a step distance of 0.5 µm were collected and single planes or maximum intensity projections were generated with Zeiss ZEN Confocal Software (Carl Zeiss MicroImaging GmbH Jena Germany).

### Flow Cytofluorimetric Analysis of FPRs Expression

WS1 cells were cultured in 100 mm Petri dishes until reaching 80% confluency. Once obtained the optimal confluency, cells were treated as previously described [17]. Briefly, WS1 cells were harvested and centrifuged at 30000×g for 5 minutes. The pellets were then incubated on ice for 1 h in 100 µl of PBS 1× containing a primary polyclonal antibody against FPR or a primary monoclonal antibody against FPR2 (1∶1000; Genovac and R&D Systems respectively). After that, WS1 cells were washed twice and incubated on ice for 1 h in 100 µl of PBS 1× containing AlexaFluor 488 anti-rabbit (1∶1000; Molecular Probes) or AlexaFluor 488 anti-mouse (1∶1000; Molecular Probes). The cells were analyzed with Becton Dickinson FACScan flow cytometer using the Cells Quest program.

### Measurement of Intracellular Ca^2+^ Signaling

Intracellular Ca^2+^ concentrations [Ca^2+^] were measured using the fluorescent indicator dye Fura 2-AM, the membrane-permeant acetoxymethyl ester form of Fura 2, as previously described [18] with minor revisions. Briefly, WS1 cells (1×10^5^/ml) were washed in (PBS) resuspended in 1 ml of Hank’s balanced salt solution (HBSS) containing 5 mM Fura 2-AM and incubated for 45 min at 37°C. After the incubation period, cells were washed with the same buffer to remove excess of Fura 2-AM and then incubated in 1 ml of buffer containing or not 0.1 mM Ca^2+^. WS1 cells were then transferred to the spectrofluorimeter (Perkin-Elmer LS-55). Treatments with ionomycin (1 mM) and/or fMLP (50 nM), Ac2-26 (1 µM), CiclosporinH (500 nM) were carried out by adding the appropriate concentrations of each substance into the cuvette in Ca^2+^-free HBSS/0.5 mM EDTA buffer.

The excitation wavelength was alternated between 340 and 380 nm, and emission fluorescence was recorded at 515 nm. The fluorescence ratio was calculated as F340/F380 nm. Maximum and minimum [Ca^2+^] were determined at the end of each experimental protocol by adding to the cells HBSS containing 1 mM ionomycin and 15 mM EDTA, respectively, according to the equation of Grynkiewicz [19].

### siRNA Oligonucleotides Preparation and Transfection

The siRNA against ANXA1 was siGENOME ON-TARGETplus SMARTpool reagent L- 040923-00-0005, 5 nmol, Human ANXA1 (Dharmacon Research, Inc., Lafayette, CO). siRNA Oligo Scrambled (SK) KROAA-006461 (sense 5′-CAGUCGCGUUUGCGACUGG-3′) (Dharmacon Research, Inc.) was used as control. WS1 cells were initially plated in LG or HG media containing 10% FBS. After 3 days, cells were washed once with PBS and transfected or not with siRNAs by Nucleofector (Amaxa, Inc., Cologne, Germany) according to the manufacturer’s instructions. At the end of a 72 h incubation period the cells were processed for Western blot analysis.

### In vitro Wound-healing Assay

WS1 cells were seeded in a 12-well plastic plate at 2×10^5^ cells per well. After the selected treatment, cells reached 100% confluency and a wound was produced at the centre of the monolayer by gently scraping the cells with a sterile plastic p200 pipette tip. In ANXA1 knock down experiments, after removing transfection medium, cells cultures were incubated in the presence or not of 25 mM of D-glucose. For neutralization experiments of cell surface ANXA1, later than removing incubation medium and washing twice with PBS1x, cell cultures were incubated with ANXA1 neutralizing antibody (1∶100; Invitrogen) or with Rabbit pre-immune IgG (1∶100; Sigma Aldrich) or in culture medium as control in presence or not of 25 mM D-glucose. For pharmacologic experiments, after removing incubation medium and washing with PBS, cell cultures were incubated in the presence of fMLP (50 nM; Sigma-Aldrich), Ac2-26 (1 µM; Tocris), Ciclosporin H (500 nM; Alexis) or in GM as control in presence or not of 25 mM of D-glucose. The wounded cell cultures were then incubated at 37°C in a humidified and equilibrated (5% v/v CO_2_) incubation chamber of an Integrated Live Cell Workstation Leica AF-6000 LX. A 10 × phase contrast objective was used to record cell movements with a frequency of acquisition of 10 minutes. The migration rate of individual cells was determined by measuring the distances covered from the initial time to the selected time-points (bar of distance tool, Leica ASF software). For each condition five independent experiments were performed. For each wound five different positions were registered, and for each position ten different cells were randomly selected to measure the migration distances. Statistical analysis were performed by using the Microsoft Excel™ software. Data were analyzed using unpaired, two-tailed t-test comparing two variables. Data are presented as means ± SD. Values <0.01 were considered as significant.

### Statistical Analysis

All results are mean ± SEM of 3 or more experiments performed in triplicate. The optical density of the protein bands detected by Western blotting was normalized on tubulin levels. Statistical comparison between groups were made using Bonferroni parametric test. Differences were considered significant if p<0.01.

## Results

### Expression and Localization of ANXA1 in Normal Human Skin Fibroblast Cell Line in High Glucose

**Figure 1:**
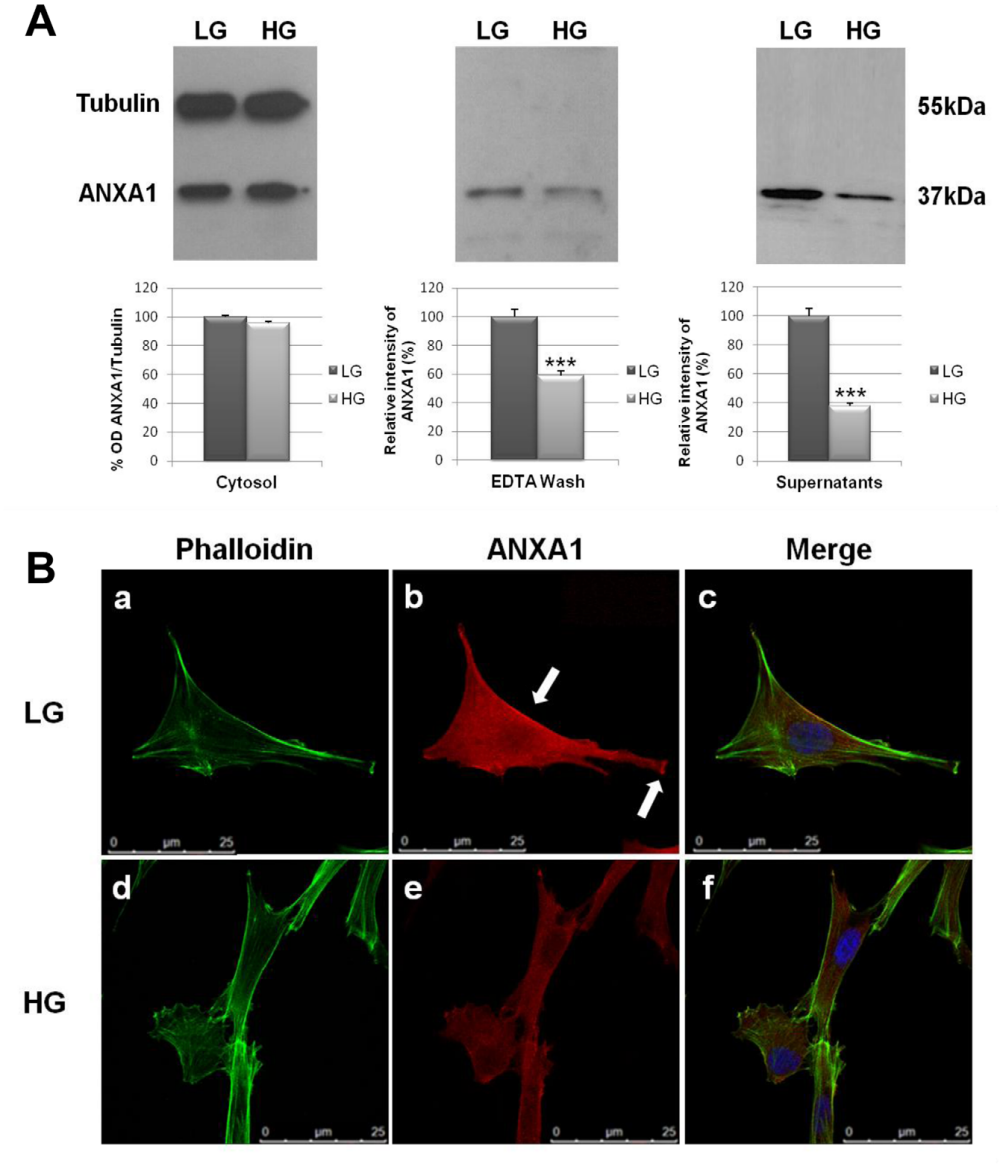
Expression and localization of ANXA1 in human skin fibroblast cells innormal and high glucose conditions. (**A**) Intracellular (Cytosol), cell surface (EDTA wash) and extracellular (Supernatants) ANXA1 expression from WS1 cells after 3 days treatment in LG and in HG conditions, was analyzed by Western blot with anti-ANXA1 antibody. The optical density of the protein bands was normalized on tubulin levels. The relative intensities of bands were determined using Gel-Doc 2000 software (BIO-RAD) and to the control band was given an arbitrary value of 100. ***p<0.001 vs LG. (**B**) Cultured human WS1 fibroblasts fixed and labeled with fluorescent antibody against ANXA1 (red) and with FITC-conjugated Phalloidin (green) in LG (a, b, c) and HG conditions (d, e, f). In normal glucose conditions ANXA1 protein is highly expressed in WS1 fibroblast cells and accumulates at the plasma membrane and at lamellipodial extrusions (arrows). An increase of glucose levels leads to a reduction of ANXA1 expression and inhibits the relocation of the protein to the plasma membrane (e). The data are representative of 5 experiments with similar results. Bar = 25 µm.

Altered expression profiles, phosphorylation, subcellular localization, and/or specific modulation of mitogenic signals are all possible mechanisms by which ANXA1 protein could explicate its biological effects [20]. Initial experiments were performed to characterize the expression of ANXA1 in normal human skin fibroblast cell line WS1. The results obtained, by Western blot analysis (Figure 1A) and immunofluorescence microscopy (Figure 1B) showed that cytosolic ANXA1 is strongly expressed in WS1 cells. ANXA1 has an important role in cell-cell communication, cell adhesion, migration and fusion. In several systems, its actions are exerted extracellularly via membrane-bound receptors on adjacent sites after translocation of protein from the cytoplasm to the cell surface [21]. Therefore, we examined the translocation of ANXA1 onto cell membrane and its secretion outside WS1 cells. Our results showed that ANXA1 translocates on WS1 cell membranes and that the protein is secreted outside the cells (Figure 1A). Immunofluorescence microscopy confirmed that ANXA1 is expressed at the cell membrane where the protein partially co-localizes with F-actin (Figure 1B) and showed that protein is enriched at the lamellipodial extrusions (Figure 1B). To determine the effects of hyperglycemia on skin fibroblasts we examined the expression and localization of ANXA1 by Western blot analysis and immunofluorescence microscopy. We treated WS1 cells with LG, or HG media for 3 days. Interestingly, analysis of ANXA1 translocation to cell membrane showed a significant reduction of the ANXA1 membrane pool in WS1 cells treated with HG compared to LG-treated cells (Figure 1A). Moreover, ANXA1 reduction observed on the cell membrane in HG-treated cells, was accompanied by lower levels of secreted ANXA1. Similar results were observed by immunofluorescence microscopy. In fact, we detected much less small immunofluorescent cytoplasmic patches and less ANXA1 on the surface of the cells in HG-treated cells (Figure 1B).

### ANXA1 is Involved in WS1 Cells Migration under High Glucose Conditions

**Figure 2:**
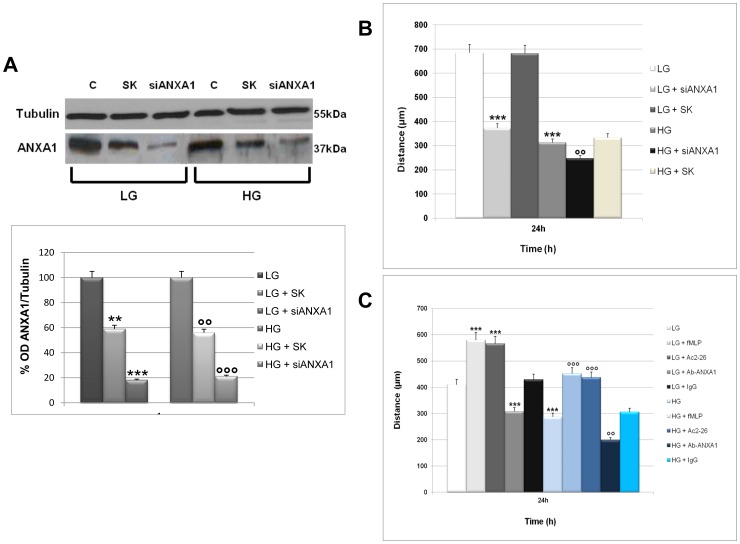
Effects of ANXA1 knock down or neutralization on WS1 cell migration. (**A**) Cell extracts from WS1 cells treated or not with siRNAs direct against ANXA1 in LG or HG conditions were analyzed by Western blot using an anti-ANXA1 antibody. The optical density of the protein bands was normalized on tubulin levels. ***<0,001, **<0,01 vs LG; °°°<0,001, °°<0,01 vs HG. (**B**) Cell migration into a scraped wound of WS1 cells treated or not with siRNAs against ANXA1 in LG or HG conditions. Results are reported as means of three experiments, measuring individual cell migrations 24 h after scraping. Bars represent standard errors. ***<0.001 vs LG; °°<0.01 vs HG. (**C**) Cell migration into a scraped wound of WS1 cells treated or not with fMLP (50 nM), Ac2-26 (1 µM) and an ANXA1 blocking antibody (1∶100) in LG or HG conditions. Results are reported as means of three experiments, measuring individual cell migrations 24 h after scraping. Bars represent standard errors. ***<0.001 vs LG; °°°<0.001, °°<0.01 vs HG.

The regulatory action of cell surface or extracellular ANXA1 has been shown to be mediated by signaling through (FPRs) [22]–[24]. In leukocytes, extracellular signals transmitted through FPRs induce polarized actin filament assembly [25]. It is therefore likely that FPR signaling also regulates cell motility by stimulating actin filament assembly and reorganization that are critical events underlying cell migration. To investigate the role of ANXA1 in WS1 cells migration, small interference (si)RNAs were used to interfere with ANXA1 expression, as described in the Materials and Methods Section. Western blot analysis showed that siRNAs directed against ANXA1 is able to reduce the expression of the protein in WS1 cells (Figure 2A). Scrambled control siRNAs had a significant effect on ANXA1 expression (Figure 2A). We treated WS1 fibroblasts with LG or HG media for 3 days, next we performed a wound-healing assay on WS1 monolayer cell line transfected with ANXA1 siRNAs. The confluent cultures were scraped to create a wound and cell migration was monitored by time-lapse video-microscopy at the site of the wound. We measured the migration distances of selected cells at different time points. Figure 2B shows a slowing down in migration speed of cells treated with ANXA1 siRNAs compared to control cells and scrambled treated cells 24 h after scraping. Moreover, a wound-healing assay on WS1 monolayer cell line transfected with ANXA1 siRNAs in HG treated cells show a significant decrease of the migration speed compared to LG or scrambled.

In order to determine whether extracellular ANXA1 is able to increase fibroblast cell migration in HG conditions, we performed a wound-healing assay on WS1 monolayer cell line in the presence of the neutralizing antibody for ANXA1. We treated WS1 fibroblasts with LG or HG media for 3 days and then 24 h in the presence of ANXA1 NH2-terminal peptide Ac2-26, FPR agonist (fMLP) and in the presence of a neutralizing antibody for ANXA1. The confluent cultures were scraped to create a wound and cell migration was monitored by time-lapse video-microscopy at the site of the wound. The results showed that Ac2-26 (1 µM) or fMLP (50 nM) are able to increase fibroblast cell migration compared to control cells 24 h after scraping. Conversely, we observed a slowing down in migration speed of cells treated with ANXA1 neutralizing antibody compared to control cells and IgG treated cells (Figure 2C). Later time points showed the closure of all control and IgG wounds, but not in the wounds of ANXA1 blocked cells (data not shown).

### Fibroblasts Express nFPRs

**Figure 3:**
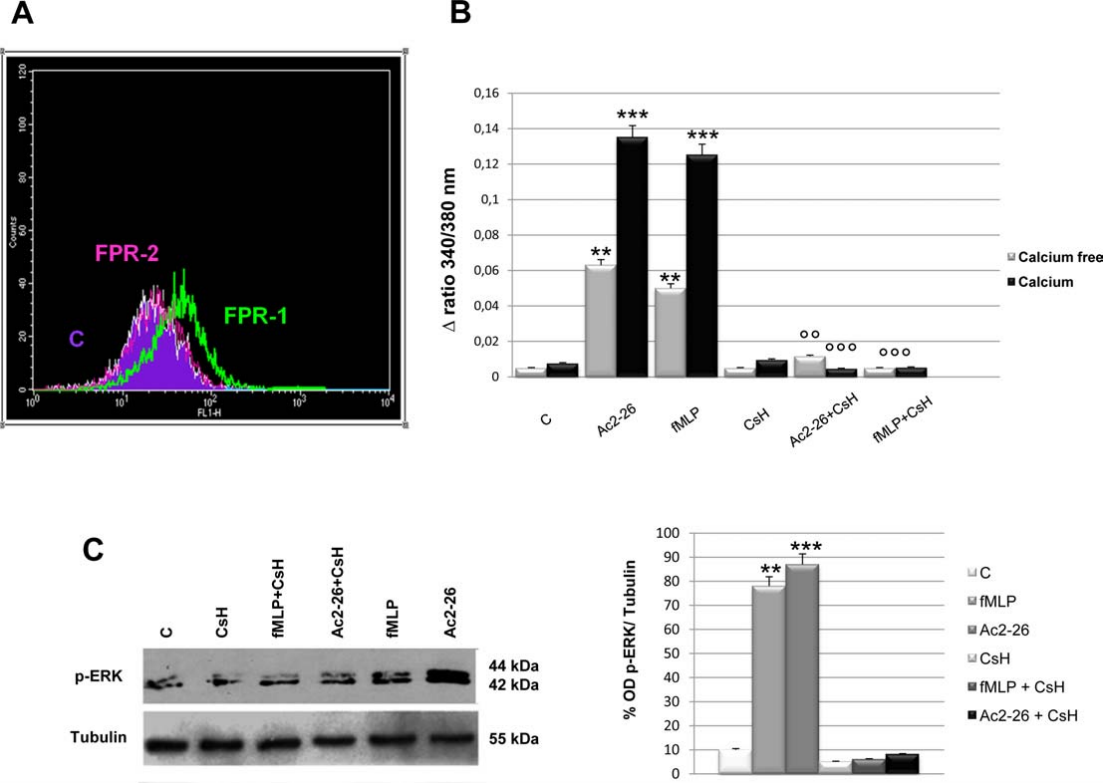
FPR expression and activation in WS1 fibroblasts. (**A**) Cell surface expression of FPR-1 and FPR-2 in human WS1 fibroblasts was analyzed by flow cytometry. FPR1 (green) is highly express in WS1 cells whereas a weak expression of FPR-2 (purple) is observed. (**B**) Effects of Ac2-26 (1 µM), fMLP (50 nM) and CsH (500 nM) on the FPR-induced rise in intracellular Ca^2+^. WS1 were treated as described in Materials and Methods. The histogram shows the ﬂuorescence ratio calculated as F340/F380 nm in the presence or in the absence of extracellular Ca^2+^. Control represents unstimulated cells. Data are means ± SEM (n = 3). *** <0.001, ** <0.01 vs corresponding controls; °°° <0.001, °° <0.01 vs corresponding Ac2-26 or fMLP. (**C**) ERK phosphorylation rate was analyzed by Western blot at different time point following Ac2-26, fMLP or CsH incubation. The Western blot is representative of the 20 minutes experimental time point. The protein levels were normalized on the α-tubulin expression. The data are representative of 3 experiments with similar results. *** <0.001; ** <0.01 vs control.

It has been shown that nFPRs could regulate cell migration through actin polymerization. Previous studies on FPR expression in human fibroblasts cell line by RT-PCR showed the expression of FPR1 and FPR2 in this cells [11]. In order to confirm these findings, we assessed the FPR expression in WS1 cells by cytofluorimetric analysis (Figure 3A), and according to previous data we found that FPR1 is highly express in WS1 cells. Conversely, we observed a weak expression of FPR-2 (Figure 3A). It is know that the interaction between ANXA1 and FPR receptors caused a series of cellular responses, such as the ERK phosphorylation and the increase of intracellular [Ca^2+^] concentration. These responses are involved in different physiological processes including growth, differentiation and cell migration, apoptosis and immune responses. To determine whether ligand binding to FPRs induces similar signal transduction in fibroblast cells, we examined the stimulated release of calcium from intracellular stores. Cells were incubated either in Ca^2+^ free medium or in medium with 0.1 mM Ca^2+^. Cells were then loaded with the fluorescent calcium indicators Fluo-2AM before stimulation with N- terminal peptide of ANXA1, Ac2-26 (1 µM) or the natural FPR agonist fMLP (50 nM). The spectrofluorimetric assay in Figure 3B shows that fMLP and peptide Ac2-26 were able to increase the mobilization of intracellular Ca^2+^ either in the presence or absence of calcium. This pattern of calcium mobilization is not observed in cells treated with the two peptides and the FPR antagonist CsH (500 nM). It has been shown that the agonist-receptor interaction involves activation of PKC and Ras, which activates the cascade of ERK1/2 MAP kinase (mitogen-activated protein kinases). The cascade of MAP kinases is involved in various processes physiological which include the growth, differentiation and cell migration, apoptosis and immune responses [26]. In order to have a further test of FPR activation by Ac2-26, we have evaluated the expression of p-ERK. WS1 cells were incubated with the peptide AC2-26 to different time (5, 10, 20 and 30 minutes) and p-ERK expression was analyzed by Western blotting. As show in Figure 3C the Ac2- 26 is able to increase expression of p-ERK after about 20 minutes of incubation. No significant increases of p-ERK expression were detected at different time points (data not shown).

### Ac2-26 Stimulates Direct Migration of WS1 Cells in High Glucose Conditions

**Figure 4:**
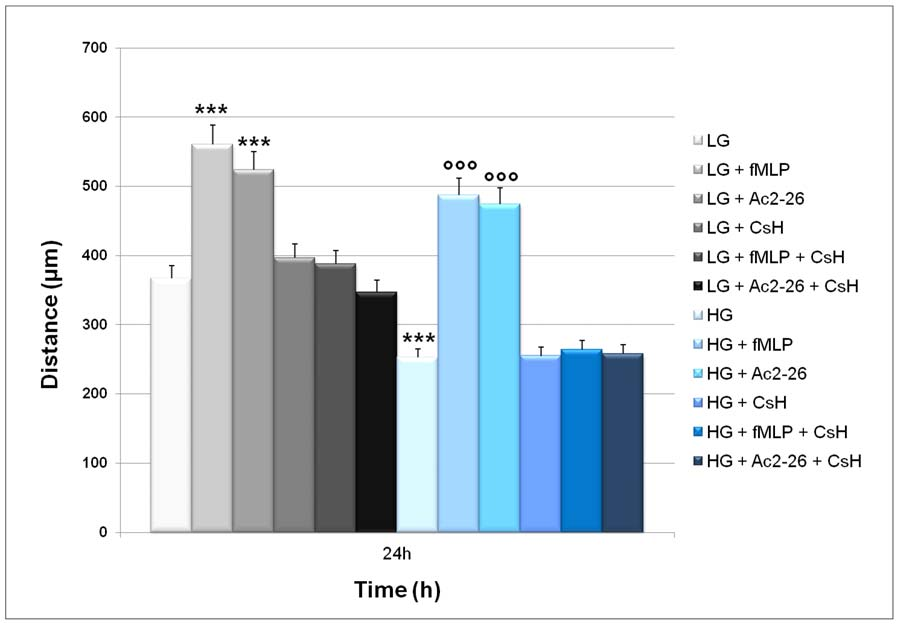
Ac2-26 stimulates direct migration of WS1 in normal and high glucose conditions. Results for control, fMLP (50 nM), Ac2-26 (1 µM), CsH (500 nM), fMLP + CsH and Ac2-26+ CsH are reported as means of three experiments, measuring individual cell migrations 24 h after scraping. Bars represent standard errors; ***<0.001 vs LG; °°°<0.001 vs HG.

To determine if ANXA1 influences fibroblast cell migration acting through FPRs, we performed a wound-healing assay on WS1 monolayer cell line in the presence of the FPR agonist fMLP, the FPR antagonist CsH, and the ANXA1-derived NH2-terminal peptide Ac2-26. We treated WS1 fibroblasts with LG or HG media, in the presence of Ac2-26, FPR agonist fMLP and in the presence of the FPR antagonist CsH. The confluent cultures were scraped to create a wound and cell migration was monitored by time-lapse videomicroscopy at the site of the wound. Results in figure 4 show an increase in migration speed of cells treated with ANXA1 N-terminal peptide Ac2-26 (1 µM) or fMLP (50 nM) compared to control cells 24 h after scraping. The stimulation of cell migration by either Ac2-26 or fMLP was inhibited by the FPR antagonist CsH (500 nM).

### Hyperglycemia Induces a Slight Increase of FPR1 Expression in WS1 Fibroblasts

**Figure 5:**
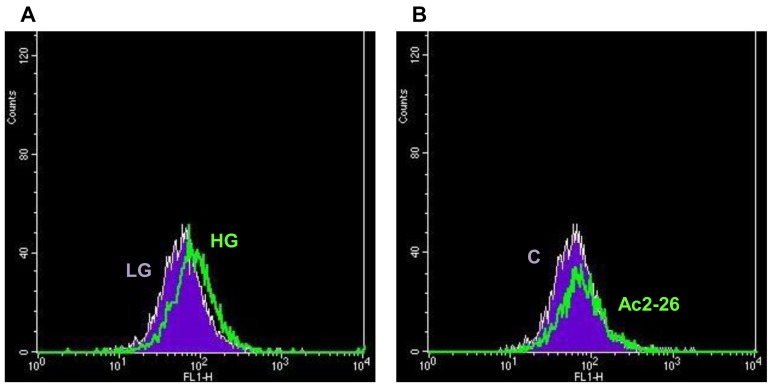
Hyperglycemia induces a slight increase of FPR1 expression in WS1 Cells. (**A**) Cell surface expression of FPR-1 in human WS1 fibroblasts incubated in LG or HG conditions was analyzed by flow cytometry. FPR1 expression is slightly increased in WS1 cells treated for 3 days in HG conditions (green) compared to LG conditions (violet). Data are representative of 3 experiments with similar results. (**B**) Cell surface expression of FPR-1 in human WS1 fibroblasts treated (green) or not (violet) with Ac2-26 peptide was analyzed by flow cytometry. The obtained results show that the Ac2-26 peptide is not able to induce any modulation of FPR1 expression. Data are representative of 3 experiments with similar results.

In order to verify whether glucose levels would affect the extent of expression of FPR1, we assessed the FPR expression in WS1 cells by cytofluorimetric analysis (Figure 5A), and we found that hyperglycemia conditions could slightly increase cell surface expression of FPR1 receptor.

Next, we investigated whether engagement of the receptor with the ANXA1 peptide might up-regulate further this GPCR (Figure 5B). Our results show that FPR1 expression was not significantly modified in WS1 cells treated by Ac2-26 peptide.

## Discussion

The process of tissue repair is characterized by a series of cellular events finely integrated through regulatory mechanisms which involving the chemotaxis and the release of cellular components within of the matrix substances which assume the role of paracrine effectors in this process. It has been shown that in the diabetic rat to which it was experimentally induced a skin lesions, normalization of glucose values with insulin therapy, leads an improvement in the healing process of ulcers, demonstrating that the diabetic condition, especially during glycometabolic failure, is associated with alterations in physiological processes of tissue repair [3], [4]. Fibroblasts are the cell component that plays a primary role in this process since their role is to wound contraction and restructuring of the extracellular matrix. Following tissue damage, fibroblasts migrate to sites inflammatory, repopulating the wound, and remodeling fibrin and collagen deposits.

Annexin-1 (ANXA1, lipocortin-1) is the first characterized member of the annexin superfamily of proteins, so called since their main property is to bind (i.e. to *annex*) to cellular membranes in a Ca^2+^-dependent manner. ANXA1 is involved in a wide range of functions both inside and outside cells such as membrane aggregation, inflammation, phagocytosis, apoptosis, proliferation, and differentiation. ANXA1 N-terminal derived peptide Ac2-26 for a long time has been used as an ANXA1 surrogate in view of its ability to replicate the anti-inflammatory effects of the parent protein [27]. Ac2-26 can activate all three human formyl peptide receptors, promoting calcium fluxes, and cell locomotion [25]. Such an effect may be part of a reparatory process as demonstrated with intestinal epithelial cells [12] where addition of the Ac2-26 peptide engaged FPRs to activate the cytoskeletal machinery favoring cell migration and repair of the epithelial monolayer. Our previous studies [13] indicate that ANXA1 could be a novel determinant for tissue repair, at least in the muscle, playing a role in stem cell (SCs in the muscle) migration and differentiation. Moreover, we found that ANXA1 N-terminal derived peptide Ac2-26 is able to induce migration of C2C12 skeletal muscle cells by acting through FPRs (unpublished).

Based on these information we evaluated the role of ANXA1 in human skin fibroblast cell line WS1 migration in normal and hyperglycemic conditions.

Initial experiments showed that WS1 cells express high levels of ANXA1. Moreover, we observed the enrichment of ANXA1 protein at cell movement structures like lamellipodial extrusions showed by our immunofluorescence experiments, suggesting a role for ANXA1 in WS1 cell motility.

Interestingly, we found significant decrease in levels of the protein in hyperglycemic conditions. In fact we examined the translocation of ANXA1 to cell membrane in WS1 cells by Western blotting and we observed lower levels of ANXA1 in both membrane pool and supernatants of WS1 cells treated with HG. This finding has been confirmed by immunofluorescence microscopy, which showed much less ANXA1 on the surface of the cells.

A possible direct association of ANXA1 with F-actin and different actin-binding proteins has been reported as important in actin polymerization and in connecting actin network to plasma membrane [28]. It was shown that hyperglycemia is able to induce cortical actin network disassembly with subsequent relocation of plasma membrane bound proteins like filamin from the cell periphery to the cytosol [29]. It is then conceivable that the ANXA1 relocation we observed in HG conditions could be due to the cortical actin disassembly following HG exposure.

Wound-healing assays using cell line transfected with ANXA1 siRNAs showed a slowing down in migration speed of cells treated with ANXA1 siRNAs confirming that ANXA1 has a role in the migration of WS1 cells. Moreover, a wound healing assay on WS1 monolayer cell line transfected with ANXA1 siRNAs in HG treated cells showed a significant decrease of the migration speed compared to LG or scrambled. Next, with the aim to verify the role in the migration of extracellular ANXA1 in HG conditions, we showed that N-terminal peptide of ANXA1 Ac2-26 is able to increase fibroblast cell migration. Conversely, we observed a progressive slowing down in migration speed of cells treated with ANXA1 neutralizing antibody. The same data were also obtained in response to conditions of hyperglycemia. The data we obtained seem to indicate the involvement of extracellular ANXA1 in the migration process of WS1 cell.

As previously mentioned the ANXA1 action is carried out through FPRs, therefore we went to investigate the expression and the activation of this receptors. In agreement with the literature [11], we have demonstrated, by flow cytometry, the expression of the receptor FPR1 in WS1 cells. Experiments on the mobilization of intracellular calcium and analysis of p-ERK expression conducted in WS1 cells, have confirmed the activity of the FPR1 following stimulation with the Ac2-26 peptide.

Finally, wound-healing assay on WS1 monolayer cell line in the presence of the well known FPR agonist fMLP, of the FPR antagonist CsH and in the presence of Ac2-26 show that ANXA1 influences fibroblast cell migration under high glucose conditions acting through FPR receptors.

Finally, cytofluorimetric analysis of FPR1 expression in WS1 cells showed that hyperglycemia conditions could slightly increase cell surface expression of this receptor. This up-regulation of FPR1 expression could explain the effect of Ac2-26 peptide in positive modulating WS1 migration despite HG conditions.

In conclusion, these data report for the first time the involvement of ANXA1 in the processes of cell migration in human skin fibroblast WS1, through interaction with FPRs in different glucose conditions. Interestingly the N-terminal peptide of ANXA1, Ac2-26 was able to stimulate direct migration of WS1 cells in high glucose treatment possibly due to the increased receptor expression observed in hyperglycemia conditions.
